# CHMMOTv1 - cardiac and hepatic multi-echo (T2^*^) MRI images and clinical dataset for Iron overload on thalassemia patients

**DOI:** 10.1186/s13104-023-06607-5

**Published:** 2023-11-16

**Authors:** Iraj Abedi, Maryam Zamanian, Hamidreza Bolhasani, Milad Jalilian

**Affiliations:** 1 Department of Medical Physics, School of Medicine, Isfahan University of Medical Sciences, Isfahan, Iran; 2 Department of Computer Engineering, Islamic Azad University Science and Research Branch, Tehran, Iran; 3 Department of Neuroscience, Neuroimaging and Addiction Studies, School of Advanced Technologies in Medicine, Tehran University of Medical Sciences, Tehran, Iran

**Keywords:** Cardiac MRI, Dataset, Thalassemia, T2* magnetic resonance imaging, Iron overload, Hepatic system

## Abstract

**Introduction:**

Regarding deep learning networks in medical sciences for improving diagnosis and treatment purposes and the existence of minimal resources for them, we decided to provide a set of magnetic resonance images of the cardiac and hepatic organs.

**Database description:**

The dataset included 124 patients (67 women and 57 men) with thalassemia (THM), the age range of (5–52) years. Patients were divided into two groups: with follow-up (1–5 times) at time intervals of about (5–6) months and without follow-up. T2* and, R2* values, the results of the Cardiac and Hepatic overload report (normal, mild, moderate, severe), and laboratory tests including Ferritin, Bilirubin (D, and T), AST, ALT, and ALP levels were provided as an Excel file. Also, the details of the patients’ Echocardiogram data have been made available. This dataset CHMMOTv1) has been published in Mendeley Dataverse and also is accessible through the web at: http://databiox.com.

## Introduction

Thalassemia (THM) is one of the most inherited hemoglobinopathies and the most common monogenic disorder worldwide. Every year, approximately 300–400 thousand fetal-affected types with anemia are born [1, 2]. Due to the location of the mutation in the hemoglobin chain, it is classified into two groups: α-thalassemia (α-THM) as the fetal type and β-thalassemia (β-THM) divided into minor and major types [1, 3]. The need for frequent blood transfusions to relieve the effects of anemia can lead to complications, such as iron overload (IrO) in the cardiac, hepatic, and endocrine glands. Regular chelation therapy and early diagnosis are important for dangerous complications of iron in the liver such as hepatomegaly (fibrosis and cirrhosis), cardiac failure, systolic and/or diastolic left ventricle (LV) dysfunction, pulmonary hypertension (PHT), and arrhythmia leading to death [4].

Methods include a hepatic biopsy, serum iron and ferritin levels, transferrin saturation, and magnetic resonance imaging (MRI) were used to calculate IrO. Although serum ferritin level estimation is inexpensive and the most accessible method for assessing the body’s iron concentration because it shows the short-term total iron of the body, this test has low precision [5, 6]. Non-transferrin-bound iron and redox-fraction-sensitive plasma iron are also very complex, and because their results are affected by the last treatment injection, they are less valid than other tests [7]. The biopsy method is considered the “Gold standard’’, but is an invasive and dangerous method, especially for the cardiac, and has serious errors in evaluation [8, 9]. Therefore, MRI-T2* is considered a reliable and standard method for evaluating IrO in cardiac and hepatic organs [10, 11].

Magnetic resonance T2-star (T2* MRI) and T2 (T2 MRI) are methods used to assess the enhancement of tissue relaxation caused by high molecular weight iron complexes such as ferritin and hemosiderin [12]. In these methods, iron levels are obtained using different echo times (TEs); the faster the curve decreases (the lower the T2* and T2 values), the more iron there is in the tissue, and the darker the image [13, 14]. Breath-hold can be used in T2* relaxometry, while it is challenging in T2 relaxometry, especially if a “pure SE” approach is followed as in Ferriscan (add reference here) [15]. In addition to, studies have shown that T2* provides the most excellent estimates up to a 24 weeks interval [16, 17]. Tissue relaxation can be also expressed as relativity rates: R2 (1/T2) or R2* (1/T2*) [18].

The ability of artificial intelligence to facilitate diagnosis and treatment procedures and the need for data related to the same domain for training neural networks highlight the importance of preparing datasets with the aim of speeding up the work. This study is the first to simultaneously prepare a set of cardiac MR images (CMR) and hepatic MR images (HMR) for the analysis and image processing of THM patients.

## Database description

All T2* MR images were obtained using GE Healthcare (Waukesha, USA). Cardiac gating MRI examination was performed using a single mid-papillary ventricular short-axis slice in the supine position with a single breath-hold using a torso phased-array body coil, and a multi-echo gradient-echo sequence (12 echoes) was obtained. The field of view (FOV) in CMR extends caudally from the carina to the lower renal pole (40 × 40) cm, matrix size (128 × 116), slice thickness 10 mm, 12 different TEs (between 1.8, and 17.9) ms, TR (31.3) ms, and bandwidth 1562 Bw/pixel. A cardiac T2* value calculated by T2 mapping with a time of more than 20 ms is considered normal for Iron Overload (IrO). A T2* value between 15 and 20 ms is considered mild, between 10 and 15 ms is moderate, and less than 10 ms is indicative of severe myocardial siderosis.

For the HMR, a single breath-hold technique using a multi-echo gradient-echo in 12 different TEs (1.02, to 15.06) ms, FOV (40 × 40) cm, matrix size (128 × 116), voxel size should have 3.1 * 3.4 mm in-plane resolution, slice thickness 8 mm, 120 ms, and bandwidth 1736 BW/pixel. The flip angles for both CMR and HMR were 20^0^. Hepatic T2* values of less than 30 ms indicate hepatic abnormal IrO: mild (> 6/2) ms, moderate (3.1–5.2) ms, severe (2.1-3. 1) ms.

To determine iron concentration, we used a GE workstation software tool workstation following acquisition using a multi-echo T2 star. To eliminate bias and computation errors, we followed these steps:

1. The intensity correction filter was disabled prior to data acquisition.
2. Exclude vein, artery, and hepatic duct for computation.
3. A radiologist marked Regions of Interest (ROIs) on the mid-liver slice and mid-ventricular short-axis slice of the generated R2* map and computed the average R2* value for liver and cardiac, respectively.
4. After the third step, the R2* value was converted to liver iron concentration (LIC) using the formula LIC = 0.0254 × (R2*) + 0.202 as described by Wood et al. [19].
5. For calibration, we calculate mean liver iron concentration (LIC) using liver R2 values and a calibration curve determined through a needle biopsy as described by Tim St Pierre et al. [13].

The current dataset included 210 MRI image files (every file different series) of 124 patients with THM, including 67 women and 57 men age range (5–52) years. The data were divided into two groups with 75 patients with follow-up (Between 1 and 5 times) in time intervals of about (5–6) months and 48 patients without follow-up. The image format was DICOM, with a 16-bit grayscale resolution of (192 × 256) pixels. After anonymization to protect the security of the patients, the image files were stored in RAR files.

**Fig. 1:**
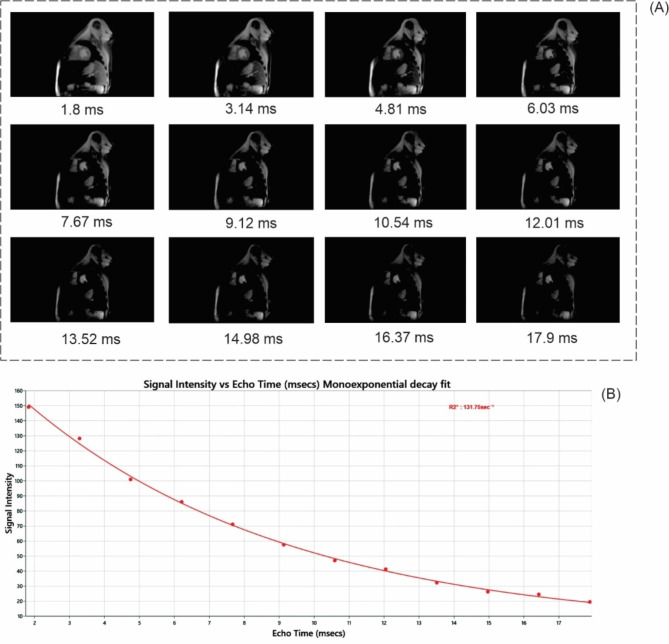
A sample of the MRI images is related to a one patient with severe thalassemia (THM) (A), and curve shows change of signal intensity versus time of echo values (B)

In addition, an Excel file containing T2* and R2* values and a report on cardiac and hepatic IrO of the patients (normal, mild, moderate, severe) was provided. Figure 1 shows a sample of images from the current dataset.

**Fig. 2:**
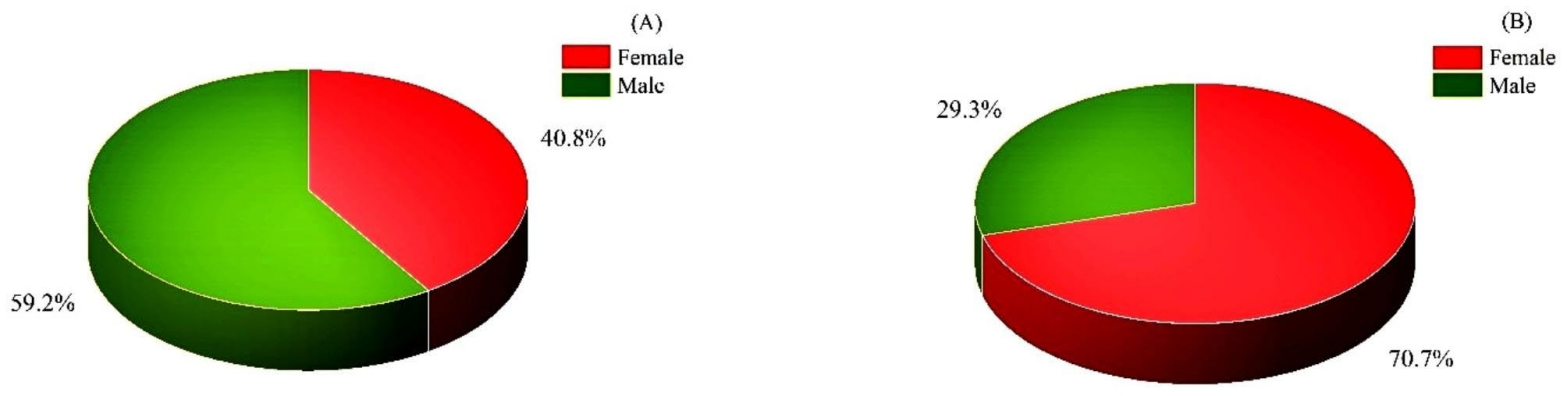
Distribution of the number of men and women in the two categories with (A) and without follow-up (B)

**Fig. 3:**
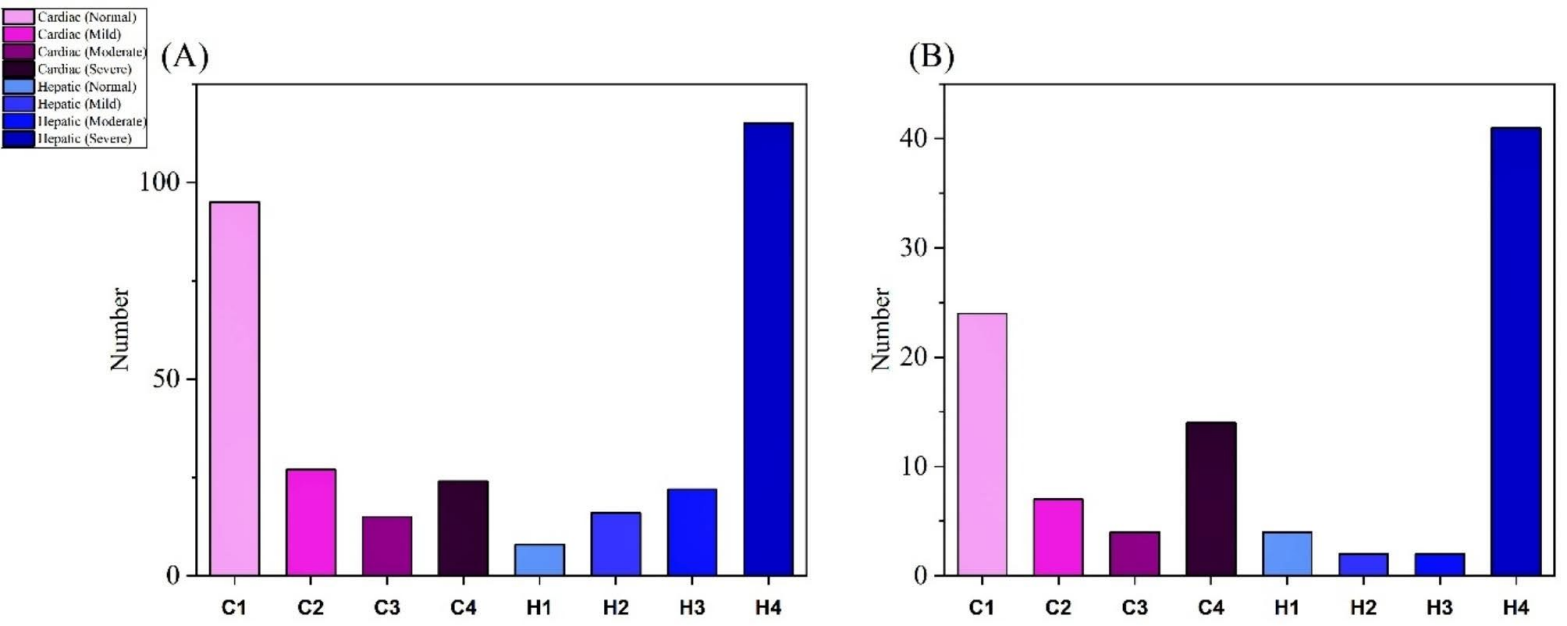
In part (A), the Cardiac and Hepatic situation of patients with Follow-up and in part (B) of patients without Follow-up (The dotted left part in both bar plots is for the Cardiac and the right part for the Hepatic organ, which correspond to the reported normal, mild, moderate, severe, respectively)

The sex separation of the patients as well as their cardiac and hepatic IrO conditions are shown in detail in Figs. 2 and 3. The patient’s blood test results, including Ferritin, Bilirubin (D and T), aspartate aminotransferase (AST), alanine aminotransferase (ALT), and alkaline phosphatase (ALP) levels, were provided in an Excel file.

To date, the only dataset provider study for patients with THM was conducted by Shiae et al. in Mashhad, Iran, between February 2016 and January 2019, in the form of open-source CMR images of 50 subjects, including 37 THM patients and 13 healthy subjects, with clinical and echocardiographic data, such as clinical signs of heart failure, shortness of breath, decreased activity, hand and foot swelling, round the eye and chest pain, and arrhythmia. All images were 16-bit grayscale with a resolution of (192 × 256) pixels, stored in DICOM format, and finally compressed and saved in RAR format [20].

However, present dataset including a larger number of patients, has two sections with and without Follow-up to perform more various studies such as disease prediction. Also, we mentioned the status report of hepatic IrO in the dataset, which can be used in some hepatic studies.

The provision of Echocardiography data of patients is also another noteworthy point of this study, which is in Excel format in dataset. The details of this file include “Ejection Fraction (EF), Left Ventricular Size (LVSize), (TGR), Pulmonary Artery Pressure (PAP), Tricuspid Annular Plane Systolic Excursion (TAPSE), and ECG Report " which does not include the with Follow-up section of the patients and is for Without Follow-up and the first stage with Follow-up.

## Data Availability

The data described in this dataset including Excel and MRI images as RAR fill can be accessed on the Mendeley data site (https://data.mendeley.com/datasets/3d8bwn2fsh/1).

## Abbreviations

IrO: Iron overload
α-THM: α-thalassemia
β-THM: β- thalassemia
PHT: pulmonary hypertension
MRI: magnetic resonance imaging
CMR: cardiac MR
HMR: hepatic MR
Echo: echocardiography, EF:Ejection Fraction
LVSize: Left ventricular Size
PAP: Pulmonary Artery Pressure
TAPSE: Tricuspid Annular Plane Systolic Excursion

## References

[1] Origa R (2017). β-Thalassemia. Genet Med.

[2] Shahnavazi A, Abdi N, Allahyari E, Bakhshi F, Peighan A (2016). Effectiveness of eye movement desensitization and reprocessing on anxiety in children with thalassemia in a 12-month follow up. Armaghane Danesh.

[3] Farashi S, Harteveld CL (2018). Molecular basis of α-thalassemia. Blood Cells Mol Dis.

[4] Meloni A, Puliyel M, Pepe A, Berdoukas V, Coates TD, Wood JC (2014). Cardiac iron overload in sickle-cell Disease. Am J Hematol.

[5] Porter JB, Elalfy M, Taher A, Aydinok Y, Lee SH, Sutcharitchan P (2017). Limitations of serum ferritin to predict liver iron concentration responses to deferasirox therapy in patients with transfusion-dependent thalassaemia. Eur J Haematol.

[6] Puliyel M, Sposto R, Berdoukas VA, Hofstra TC, Nord A, Carson S (2014). Ferritin trends do not predict changes in total body iron in patients with transfusional iron overload. Am J Hematol.

[7] de Swart L, Hendriks JC, van der Vorm LN, Cabantchik ZI, Evans PJ, Hod EA (2016). Second international round robin for the quantification of serum non-transferrin-bound iron and labile plasma iron in patients with iron-overload disorders. Haematologica.

[8] Baksi AJ, Pennell DJ (2014). Randomized controlled trials of iron chelators for the treatment of cardiac siderosis in Thalassaemia major. Front Pharmacol.

[9] Wahidiyat PA, Liauw F, Sekarsari D, Putriasih SA, Berdoukas V, Pennell DJ (2017). Evaluation of cardiac and hepatic iron overload in Thalassemia major patients with T2* magnetic resonance imaging. Hematology.

[10] Siri-Angkul N, Chattipakorn SC, Chattipakorn N (2018). Diagnosis and treatment of cardiac iron overload in transfusion-dependent thalassemia patients. Expert Rev Hematol.

[11] Golfeyz S, Lewis S, Weisberg IS (2018). Hemochromatosis: pathophysiology, evaluation, and management of hepatic iron overload with a focus on MRI. Expert Rev Gastroenterol Hepatol.

[12] Cheng HL, Holowka S, Moineddin R, Odame I (2012). Liver iron overload assessment by T *2 magnetic resonance imaging in pediatric patients: an accuracy and reproducibility study. Am J Hematol.

[13] St Pierre TG, Clark PR, Chua-anusorn W, Fleming AJ, Jeffrey GP, Olynyk JK (2005). Noninvasive measurement and imaging of liver iron concentrations using proton magnetic resonance. Blood.

[14] St Pierre TG, El-Beshlawy A, Elalfy M, Al Jefri A, Al Zir K, Daar S (2014). Multicenter validation of spin-density projection-assisted R2-MRI for the noninvasive measurement of liver iron concentration. Magn Reson Med.

[15] Kaltwasser JP, Gottschalk R, Schalk KP, Hartl W (1990). Non-invasive quantitation of liver iron-overload by magnetic resonance imaging. Br J Haematol.

[16] Wood JC, Zhang P, Rienhoff H, Abi-Saab W, Neufeld EJ (2015). Liver MRI is more precise than liver biopsy for assessing total body iron balance: a comparison of MRI relaxometry with simulated liver biopsy results. Magn Reson Imaging.

[17] Menacho K, Abdel-Gadir A, Moon JC, Fernandes JL (2019). T2* mapping techniques: Iron Overload Assessment and other potential clinical applications. Magn Reson Imaging Clin N Am.

[18] Labranche R, Gilbert G, Cerny M, Vu K-N, Soulières D, Olivié D (2018). Liver iron quantification with MR imaging: a primer for radiologists. Radiographics.

[19] Wood JC, Enriquez C, Ghugre N, Tyzka JM, Carson S, Nelson MD (2005). MRI R2 and R2* mapping accurately estimates hepatic iron concentration in transfusion-dependent thalassemia and sickle cell Disease patients. Blood.

[20] Shiae Ali E, Bakhshali MA, Shoja Razavi SJ, Poorzand H, Layegh P (2021). Cardiac MR images of Thalassemia major patients with myocardial iron overload: a data note. BMC Res Notes.

